# Benign Neoplasms in Biallelic Nth-Like DNA Glycosylase 1-Associated Tumor Syndrome: Expanding the Clinical Phenotype

**DOI:** 10.14309/crj.0000000000002199

**Published:** 2026-06-18

**Authors:** Daniele Macchi, Eleonora Cortellazzi, Vera Uliana, Enrico Ambrosini, Francesca Negri, Patrizia Caggiati, Antonio Percesepe, Luigi Laghi, Stefano Kayali

**Affiliations:** 1 Department of Medicine and Surgery, University of Parma, Parma, Italy; 2 Medical Genetics, Parma University Hospital, Parma, Italy; 3 Gastroenterology and Endoscopy Unit, Parma University Hospital, Parma, Italy; 4 Laboratory of Molecular Gastroenterology, Humanitas Clinical and Research Centre, Milan, Italy

**Keywords:** *NTHL1*-associated-tumor syndrome, schwannoma, hemangioma, benign neoplasms, biallelic mutation

## Abstract

Nth-like DNA glycosylase 1-associated tumor syndrome (NATS) is a rare autosomal recessive cancer predisposition syndrome linked to colorectal, endometrial and ovarian cancers. Given its recent description in 2015, the full spectrum of benign manifestations remains uncertain. We describe a 53-year-old man with a biallelic pathogenic Nth-Like DNA Glycosylase 1 variant, c.244C>T(p.Gln82*), duodenal and colonic adenomas, and an unusual aggregation of benign neoplasms, including multiple oral, hepatic, splenic, and vertebral hemangiomas, an esophageal lipoma, schwannomas, and meningiomas. This represents the first report of schwannomas and oral or vertebral hemangiomas in biallelic NATS, expanding the phenotypic spectrum and supporting consideration of NATS in patients with colorectal polyposis and clustered benign neurovascular tumors.

## INTRODUCTION

Nth-like DNA glycosylase 1 (*NTHL1*) is a tumor suppressor gene encoding a DNA glycosylase involved in the base excision repair pathway, a key mechanism for maintaining genomic integrity and preventing tumorigenesis.^1^ Biallelic germline mutations in *NTHL1* cause an autosomal recessive tumor predisposition syndrome known as *NTHL1*-associated tumor syndrome (NATS), first described in 2015.^2,3^ To date, an increasing number of patients with germline pathogenic variants (PV) in *NTHL1* have been reported in the literature, with most cases developing at least 1 malignancy.^4^ NATS is associated with the development of duodenal and colorectal polyps; it also predisposes to gastrointestinal and extra-gastrointestinal tumors. The most frequently reported tumors include colorectal cancer (50.6%), breast cancer (32.5%), meningiomas (18.5%), and endometrial cancer (10.4%).^5,6^ NATS includes other cancers such as duodenal cancer, urothelial carcinoma of the bladder, unspecified brain tumors, basal cell carcinomas, head and neck squamous cell carcinomas, and hematologic malignancies. Benign neoplasms have also been observed, including ovarian and hepatic cysts, skin hemangiomas, intradermal nevi, seborrheic keratosis, and breast papillomas.^5^

Despite the increasing number of cases reported in the literature, information on NATS remains limited and the spectrum of its clinical manifestations is not yet fully defined. Given the wide phenotypic variability of the syndrome, extraintestinal manifestations require further exploration. Here, we present the case of a 53-year-old man with a biallelic PV in *NTHL1* who developed duodenal and colonic polyps, as well as hemangiomas, schwannomas, and meningiomas.

## CASE REPORT

**Figure 1.**
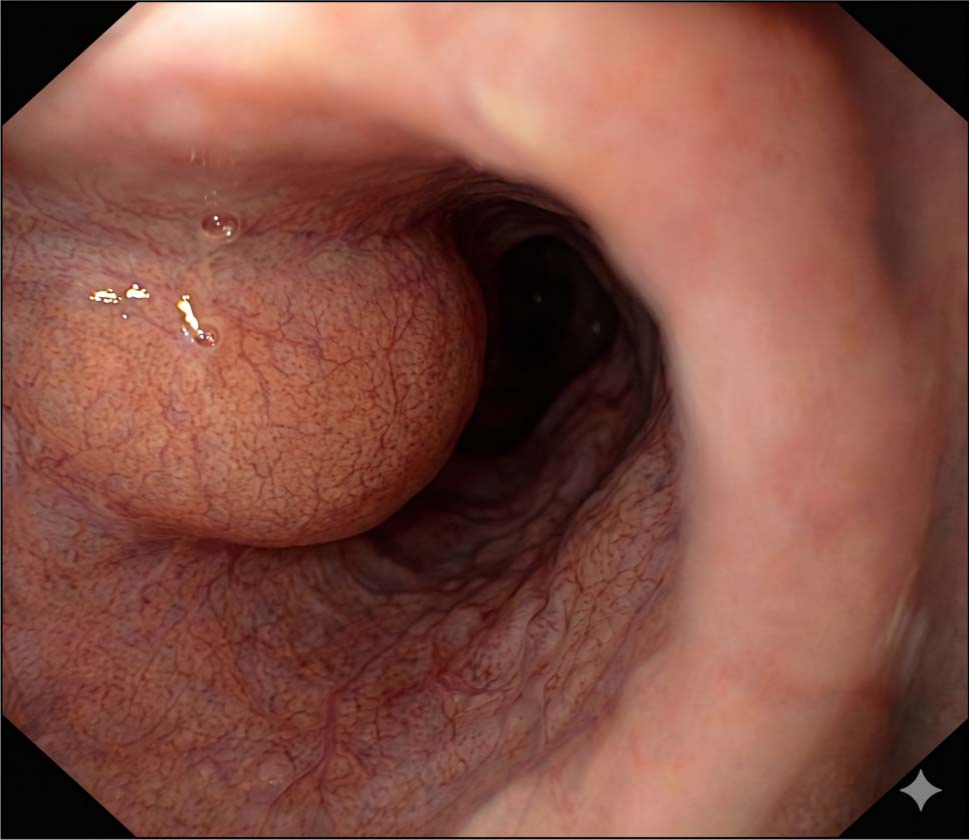
Esophageal lipoma, endoscopic image.

**Figure 2.**
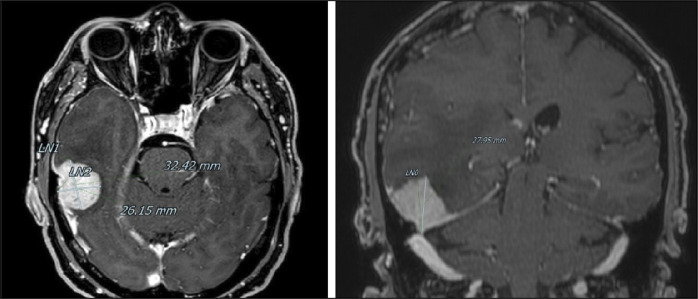
Atypical meningioma in the right temporal lobe at magnetic resonance imaging, axial, and coronal images.

A 53-year-old White man was referred to genetic evaluation due to a personal history of colonic adenomatous polyps, multiple hemangiomas, and schwannomas. Since birth, he had a cutaneous hemangioma of the lateral cervical region, which was surgically removed, and multiple oral cavity hemangiomas that required several orthodontic procedures, with stabilization of the oral condition by 18 years of age. He remained asymptomatic until 45 years when, after prolonged diarrhea and a positive fecal immunochemical test, he underwent colonoscopy, which revealed 27 polyps, including tubular and tubulovillous adenomas with low-grade dysplasia (LGD). In the same year, he underwent total colectomy for polyposis' management; histopathological analysis of the surgical specimen confirmed multiple tubular and tubulovillous adenomas with LGD. Preoperative abdominal computed tomography revealed 3 capillary and 1 cavernous hemangiomas of the liver and a splenic hemangioma. One year later, during a follow-up esophagogastroduodenoscopy, a 25-mm tubulovillous adenoma with LGD in the third portion of the duodenum and an esophageal lipoma were resected (Figure 1). At 53 years, owing to symptoms of spinal cord compression, he underwent surgery, which revealed a conventional schwannoma at T12–L1 level. Magnetic resonance imaging revealed additional lesions at the C7-T1 level, the right supratentorial region, the basal cisterns, and the left falcotentorial angle, compatible with schwannomas and initially managed conservatively. The following year, due to an increase in size of the lesions at follow-up, he underwent resection of an atypical meningioma (World Health Organization grade 2) in the right temporal region (Figure 2). Six months later, a meningothelial meningioma (World Health Organization grade 1) at the C7 level was removed. One year later he underwent Gamma Knife radiosurgery for recurrence. In the same year, he was diagnosed with a fusocellular neoplasm with a hemangiopericytoma-like pattern involving the sphenoid sinus with bone infiltration. At the time of this report, the lesion was under evaluation for definitive management. Furthermore, at 54 years, vertebral hemangiomas at the T7 level were found.

Regarding family history, he reported that a maternal aunt had an unspecified intestinal lesion and that his maternal grandmother was diagnosed with colorectal cancer at 74 years. He has no siblings, and no additional relevant family history was reported.

On physical examination, the patient presented with mild macrocephaly, melanocytic macules in the perioral region, without cutaneous neurofibromas.

Based on the clinical history and examination findings, a first genetic analysis was performed using a panel targeting the *APC*, *MUTYH*, *STK11*, and *PTEN* genes, with negative results. Given the complex phenotype, a next-generation sequencing panel for schwannomatosis and polyposis was performed on peripheral blood, identifying the homozygous variant NM_002528.7: c.244C>T (p.Gln82) in the *NTHL1* gene, establishing the diagnosis of NATS. The variant was classified as pathogenic according to American College of Medical Genetics and Genomics criteria^7^: PVS1 (null variant), PM2 (low frequency), PP1 (cosegregation), and PP5 (reported pathogenic). Unfortunately, no relatives were available for testing. After colectomy, the patient remains under regular surveillance, including periodic gastrointestinal endoscopy for adenomas. A summary of the patient's clinical course, including tumor spectrum, timing, and management, is presented in Table 1.

**Table 1.** Summary of clinical course and tumor spectrum

Age (yr)	Clinical findings	Location	Management
0–18	Cutaneous and oral hemangiomas	Cervical region, oral cavity	Surgical removal and orthodontic procedures
45	Multiple colorectal adenomas (27)	Colon	Total colectomy
45	Hepatic and splenic hemangiomas	Liver, spleen	Conservative management
46	Duodenal adenoma; esophageal lipoma	Duodenum, esophagus	Endoscopic resection
53	Schwannoma; multiple additional lesions	T12–L1; C7–T1, intracranial sites	Surgical resection; initial conservative management
54–55	Meningiomas (WHO grade 1 and 2) with recurrence	Intracranial, C7	Surgical resection; Gamma Knife
55	Fusocellular neoplasm (hemangiopericytoma-like)	Sphenoid sinus	Under evaluation
55	Vertebral hemangioma	T7	Conservative management
55	Genetic diagnosis	—	Biallelic *NTHL1* pathogenic variant (NATS)

NATS, *NTHL1*-associated tumor syndrome; WHO, World Health Organization.

## DISCUSSION

To date, only one patient with a heterozygous germline *NTHL1* variant has been reported to develop an arm schwannoma, a spinal schwannoma, and a hepatic hemangioma.^8^ A second heterozygous carrier developed spinal nerve-sheath tumors and spinal hemangiomas.^9^ To the best of our knowledge, no cases have been reported of patients with biallelic mutations of *NTHL1* experiencing schwannomas, and neither oral nor vertebral hemangiomas nor esophageal lipomas have been previously described in individuals carrying heterozygous or biallelic *NTHL1* variants. The c.244C>T (p.Gln82*) PV is the most frequently reported *NTHL1* variant in the literature.^7^ Nevertheless, several manifestations observed in our patient have not been previously reported. The identification of a sphenoid sinus fusocellular neoplasm with a hemangiopericytoma-like pattern is noteworthy. This morphology can be seen in tumors within the spectrum of solitary fibrous tumors (SFT), although a definitive classification cannot be established in the absence of confirmatory testing. This finding may further support a broader mesenchymal tumor spectrum in *NTHL1*-associated tumor syndrome. The coexistence of additional, previously unreported lesions in this case suggests that NATS may display broader phenotypic variability than currently recognized. Exome sequencing did not identify additional pathogenic variants, including in NF2, SMARCB1, or LZTR1, making alternative diagnoses such as neurofibromatosis type 2 or schwannomatosis less likely, although not completely excluded.^10^ The contribution of other genetic modifiers, such as structural or noncoding variants, cannot be entirely ruled out, although this is considered unlikely.^11,12^ This case highlights the need for further studies to better define the phenotypic boundaries and pathogenic mechanisms of NTHL1-associated tumor syndrome.

## DISCLOSURES

Author contributions: D. Macchi and E. Cortellazzi: Conceptualization, Data Curation, Writing—Original Draft. V. Uliana, Enrico Ambrosini, P. Caggiati, F. Negri, A. Percesepe: Investigation, Methodology, Formal analysis. L. Laghi: Methodology, Validation, Writing—Review & editing. S. Kayali: Methodology, Validation, Supervision, Writing—Review & editing, and is the article guarantor.

Financial disclosure: None to report.

Informed consent was obtained for this case report.

## ABBREVIATIONS:

ACMG: American College of Medical Genetics and Genomics
APC: adenomatous polyposis coli (gene)
BER: Base Excision Repair
LGD: low-grade dysplasia
LZTR1: leucine zipper like transcription regulator 1 (gene)
MRI: magnetic resonance imaging
MUTYH: MutY DNA glyglycosylase (gene)
NATS: NTHL1-associated tumor syndrome
NF2: neurofibromin 2 (gene)
NGS: next-generation sequencing
NTHL1: Nth-Like DNA glyglycosylase 1 (gene)
PM2: pathogenic moderate 2 (ACMG variant classification criterion)
PP1: pathogenic supporting 1 (ACMG variant classification criterion)
PP5: pathogenic supporting 5 (ACMG variant classification criterion)
PTEN: phosphatase and tensin homolog (gene)
PV: pathogenic variant
PVS1: pathogenic very strong 1 (ACMG variant classification criterion)
SFT: solitary fibrous tumor
SMARCB1: SWI/SNF related, matrix associated, actin dependent regulator of chromatin, subfamily B, member 1 (gene)
STK11: serine/threonine kinase 11 (gene)
WHO: World Health Organization
